# Sharing for Caring? A Patients’ and Clinicians’ View on Handling Personal Medical Data in the Context of Digitization: An Exploratory Study

**DOI:** 10.3390/healthcare12202053

**Published:** 2024-10-16

**Authors:** Kevin Frank, Thorsten Mengesdorf, Marija Radić, Philipp Herrmann, Arno Appenzeller, Henrik Mucha, Berna Orak, Indra Spiecker gen. Döhmann, Stefan Rüping, Harald Burkhardt, Michaela Köhm, Stephanie Dauth

**Affiliations:** 1 Fraunhofer Institute for Translational Medicine and Pharmacology ITMP, 60596 Frankfurt, Germany; kevin.frank@itmp.fraunhofer.de (K.F.); thorsten.mengesdorf@itmp.fraunhofer.de (T.M.); michaela.koehm@itmp.fraunhofer.de (M.K.); 2 Fraunhofer Cluster of Excellence Immune-Mediated Diseases CIMD, 60596 Frankfurt, Germany; 3 Fraunhofer Center for International Management and Knowledge Economy IMW, 04109 Leipzig, Germany; marija.radic@imw.fraunhofer.de (M.R.); philipp.herrmann@imw.fraunhofer.de (P.H.); 4 Vision and Fusion Laboratory (IES), Karlsruhe Institute of Technology (KIT), 76131 Karlsruhe, Germany; arno.appenzeller@kit.edu; 5 Hochschule der Medien Stuttgart, University of Applied Sciences, 70569 Stuttgart, Germany; mucha@hdm-stuttgart.de; 6 Chair of Law of Digitization, Institute for Digitization, University of Cologne, 50923 Cologne, Germany; orak@jur.uni-frankfurt.de (B.O.); i.spiecker@uni-koeln.de (I.S.g.D.); 7 Center for Critical Computational Studies, Goethe University Frankfurt a. M., 60629 Frankfurt, Germany; 8 Fraunhofer Institute for Intelligent Analysis and Information Systems IAIS, 53757 Sankt Augustin, Germany; stefan.rueping@iais.fraunhofer.de; 9 Division of Rheumatology, Goethe University Frankfurt, 60596 Frankfurt, Germany; harald.burkhardt@itmp.fraunhofer.de; 10 Division of Translational Rheumatology, Immunology-Inflammation Medicine, Goethe University Frankfurt, 60596 Frankfurt, Germany

**Keywords:** patient survey, exploratory study, data sovereignty, patient data management, AI in healthcare, patient involvement

## Abstract

Background: The healthcare sector is currently undergoing a significant transformation, driven by an increased utilization of data. In this evolving landscape, surveys are of pivotal importance to the comprehension of patient needs and preferences. Moreover, the digital affinity of patients and physicians within the healthcare system is reforming the manner in which healthcare services are accessed and delivered. The utilization and donation of data are influencing the future of medical research and treatment, while artificial intelligence (AI) is empowering patients and physicians with knowledge and improving healthcare delivery. Methods: In order to evaluate the opinions of patients and physicians regarding the management of personal health data and the functionality of upcoming data management devices in the context of healthcare digitization, we conducted an exploratory study and designed a survey. The survey focused on a number of key areas, including demographics, experience with digitization, data handling, the identification of needs for upcoming digitization, and AI in healthcare. Results: A total of 40 patients and 15 physicians participated in the survey. The results indicate that data security, timesaving/administrative support, and digital communication are aspects that patients associate with patient-friendly digitization. Based on the responses provided by physicians, it might be concluded that future digital platforms should prioritize usability, time efficacy, data security, and interoperability. Conclusions: In terms of expectations for future digital platforms, there is a notable overlap between the needs expressed by patients and those identified by physicians, particularly in relation to usability, time management, data security, and digital communication. This suggests that the requirements of different stakeholders can be combined in a future system, although individual issues may still require attention.

## 1. Introduction

The collection and analysis of patient data and related outcomes has become a fundamental component of healthcare systems around the globe [1]. The administration of surveys and the establishment of registries are therefore of paramount importance in order to obtain and analyze these datasets, thus facilitating an understanding of patient needs and preferences [2]. Patients are being increasingly encouraged to contribute (donate) their health data for research purposes [3,4]. The act of data donation gives rise to a number of significant questions pertaining to ownership, consent, and utilization of data [5]. It is essential to achieve a balance between utilization of patient data for scientific advancement and safeguarding of individual privacy [6]. In this regard, regulations such as the General Data Protection Regulation (GDPR) in Europe seek to provide a framework for the responsible use and protection of data [7]. It is imperative that any future system designed to handle patient data permits patients to oversee the management of their data in accordance with the relevant privacy regulations. Given that the digitization of healthcare has a multifaceted impact, encompassing technical innovations (such as the utilization of artificial intelligence (AI)); enhanced transparency in the protection, release, and utilization of personal data; and more rigorous informed consent requirements [8], it is crucial to engage data owners and users from the outset in the conceptualization and development of prospective systems [9]. 

Moreover, the incorporation of applications into clinical practice is vital for digital healthcare to achieve its full potential [10]. To be more precise, hospitals and care providers are already utilizing digital technologies, including AI, machine learning, smart sensors, and big data analytics, with the objective of enhancing the quality of care and operational efficiency [11]. The application of such technologies demonstrates considerable potential, ranging from relatively straightforward innovations in operational processes to the most challenging treatments of emergency patients [12]. In addition, policies and ethical guidelines for healthcare services emphasize the importance of obtaining proper informed consent, ensuring data security and management, and exercising responsible use of such technologies [13]. For a broad acceptance and targeted implementation of future systems, it is essential to involve stakeholders of (medical) data as much as possible in a transparent process. 

It is of utmost importance that clinicians are involved in the initial stages of the development of digital health solutions, as they represent the primary focus of patient care. Clinicians have access to a sufficient quantity of disparate data points. For data to be of value, they must be actionable and integrated with other relevant and contextual information [14]. In the absence of these characteristics, data are unable to be acted upon and instead contribute to the accumulation of irrelevant information, hindering the identification of solutions and resulting in the loss of potential opportunities. The sheer volume of disparate information coming from multiple sources is beyond the capacity of clinicians to absorb, interpret, and act upon [15]. The implementation of digital health solutions is currently encountering a number of challenges, one of which is the lack of interoperability between information technology systems [16,17,18,19]. Digital technology can facilitate the integration of disparate datasets in order to provide a comprehensive patient narrative, as opposed to a mere aggregation of disparate data points. The true value of digital technology lies in its capacity to combine disparate data sources with regulated solutions, including those that employ AI and algorithms, to derive insights with direct clinical implications [15,18]. It is imperative that digital solutions are integrated into existing workflows and aligned with the technology systems that are currently in use [17]. It is crucial for any novel digital tool to engage clinicians and demonstrate its value. Clinicians require assistance in offloading the burden of tasks that are tangential to the provision of care. These include prior insurance authorizations, documentation, accessing patient education, treatment information, and guideline updates. These tasks can be completed more efficiently and effectively through digital solutions which free up clinicians’ time, allowing them to focus on the provision of patient care [14,15,17,19]. 

Consequently, insights into patients’ and physicians’ perspectives on personal data, digital affinity, and future platforms are of great importance. Surveys represent a valuable instrument for the acquisition of insights into patients’ and physicians’ perspectives on personal data and their digital affinity [20,21,22,23,24].

While there is a certain amount of information available on the general concepts of data sharing, digitization, and AI in healthcare, there is a paucity of information pertaining to the views, opinions, experiences, and knowledge of patients and physicians on these topics. Furthermore, in recent years (and currently), there have been numerous initiatives aimed at digitizing the healthcare sector, including the development and implementation of digital systems. The deployment of digital systems in the healthcare sector is contingent upon their usability and acceptance by physicians and patients. Accordingly, in order to evaluate the opinions and experiences of patients and physicians regarding (1) the management of personal health data and (2) the functionality for prospective data management systems within the context of healthcare digitization, we designed a survey for patients and physicians. The survey focused on several key areas, including demographics, experience with digitization, data handling (e.g., providing data), the identification of needs for prospective digitization, and AI in healthcare. The surveys were designed by an interdisciplinary team that also included patient representatives and physicians. The surveys were distributed and completed anonymously by 40 patients and 15 physicians. 

In light of our findings, we suggest the necessity of incorporating key aspects and features into the handling of patient-related and practice-related data. By addressing these features, patients will be enabled to make informed decisions regarding the sharing of their data with other parties. The promotion of transparency and accountability within healthcare organizations with regard to data management will foster the development of trust between patients, physicians, and healthcare facilities, thereby enhancing the availability of data for the advancement of medical care.

## 2. Materials and Methods

In order to adopt a practice-oriented approach, we selected the medical sector of rheumatologic care at the University Hospital in Frankfurt, the Charité in Berlin, and the University Hospital of Erlangen for the purpose of conducting an exploratory study based on a survey of patients and physicians. 

**Figure 1:**
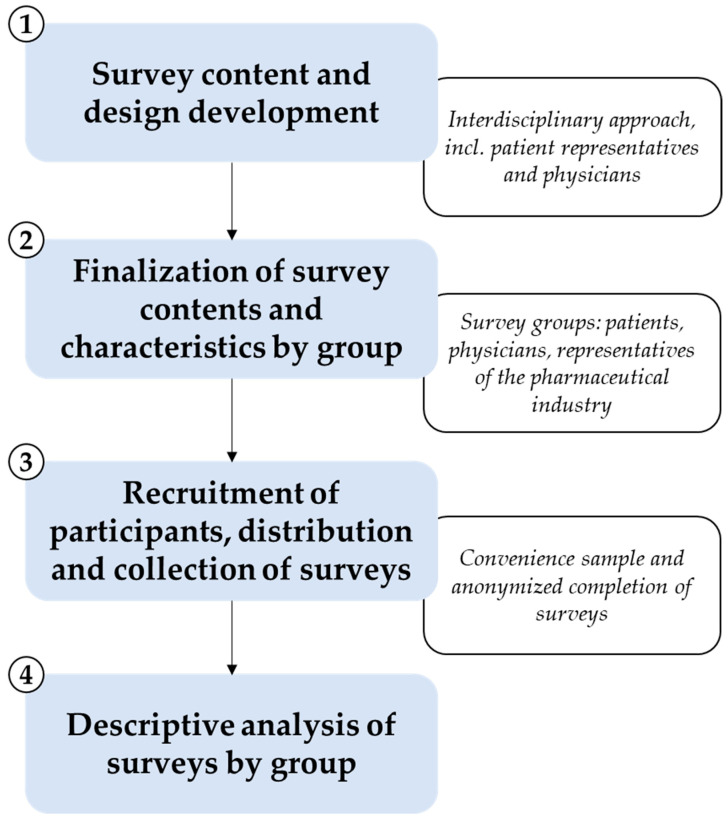
Overview of survey development, distribution, and analysis. Four main steps were undertaken for the development and implementation of the exploratory study: (1) survey content development and design of the survey with an interdisciplinary approach, including patient representatives and physicians; (2) finalization of the survey along with the characteristics and content of the surveys by group (patients, physicians, representatives of the pharmaceutical industry); (3) recruitment of participants and distribution of the survey (convenience sample) to patients, physicians, and representatives of the pharmaceutical industry and collection of completed (anonymized) surveys (incl. ethical and data protection issues); (4) descriptive analysis of surveys by group (patients, physicians, and representatives of the pharmaceutical industry).

The steps that were undertaken for the development of the survey and the implementation of the exploratory study are described in Figure 1, and the methodology employed in each of these steps will be described in detail below.

Step 1: The initial step was to design and develop the content of the surveys with the objective of gaining insights into the experiences and perspectives of patients, physicians, and representatives of the pharmaceutical industry. To facilitate enhanced communication and incorporation of the specific needs of patients [7,9], patient representatives from the Rheumaliga e.V. were invited to participate in the entirety of the survey design process. This patient advocacy group with more than 50 years of experience supports and represents 17 million people with rheumatic conditions [25]. In workshops, considerations on the individual topics for the survey (i.e., key areas that needed to be included) were discussed and supplemented by patient representatives. Additionally, physicians from the University Hospital, Goethe University (KGU) in Frankfurt were involved in the design process to reflect the everyday suitability of the survey for the daily routine of medical clinics.

Step 2: The interdisciplinary project team finalized the thematic blocks for the cross-sectional surveys, which are provided in the Supplementary Materials section.

**Figure 2:**
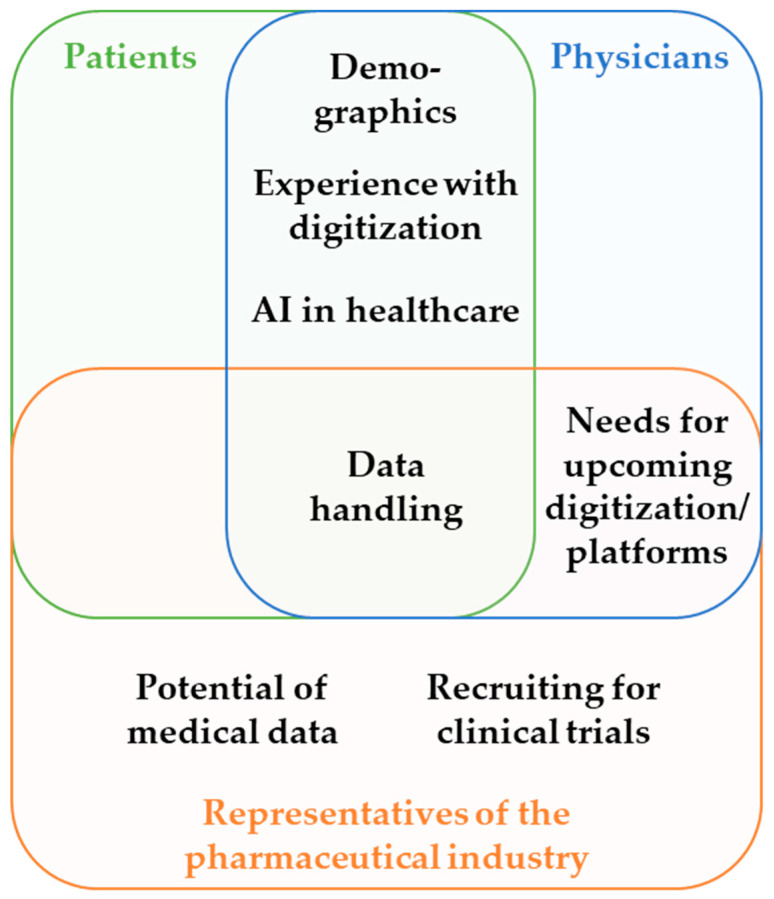
Survey content topics by group. All three groups had one topic in common: data handling. Patients and physicians shared three main topics: demographics, experience with digitization, and AI in healthcare. Physicians and representatives of the pharmaceutical industry were both asked about their needs for upcoming digitization/platforms. Topics included in the surveys for representatives of the pharmaceutical industry but not for physicians or patients were the potential of medical data and recruiting for clinical trials.

The principal areas that were identified and included in the survey are described in Figure 2.

Step 3: The surveys were distributed on a convenience basis and completed by all participating groups in an anonymous manner. Given the ongoing coronavirus pandemic at the time of survey distribution and the objective of obtaining preliminary insights into identified topics, it was determined that a convenience sample would be a more practical approach. In accordance with data protection regulation and ethical standards, the collection of purely anonymized data was conducted. The surveys did not include any personally identifying information, such as names, dates of birth, or other details that could be used to distinguish an individual.

The surveys were conducted in paper form (filled out with paper and pencil or digitally) and distributed and collected between May and October 2022. They were distributed randomly to patients attending the rheumatological study center for routine visits at the University Hospital in Frankfurt. Completion of the survey was voluntary. A total of approximately 55 patients were invited to participate in the survey. As several physicians were involved in the distribution of the survey to patients, it is not possible to provide an exact figure for the number of patients who were invited. Ultimately, 40 completed surveys were received.

The surveys were distributed to physicians at the KGU in Frankfurt, the Charité in Berlin, and the University Hospital of Erlangen, Germany, via email and also by direct distribution (paper-based). As mentioned above, participation was voluntary. In the event that a completed survey was received via email, the survey was stored on a server with restricted access, without any personal identifiers, and the email was deleted. The surveys were analyzed by a different individual to the one who received them by email. The initial invitation to participate in the study was extended to approximately 25 physicians. Additionally, these physicians were asked to disseminate the survey to their colleagues. Ultimately, 15 completed surveys were received. 

Surveys were distributed to representatives of the pharmaceutical industry by email. The initial invitation to participate in the study was extended to approximately 15 representatives of the pharmaceutical industry. Additionally, these representatives were asked to disseminate the survey to their colleagues. Ultimately, three completed surveys were received. The process of saving the surveys and analyzing them was the same as for the physicians. 

Step 4: The results of the surveys were subjected to descriptive analysis and subsequently presented. In order to test whether the differences between exploratory subgroups were statistically significant, we employed Fisher’s exact test (significance level at 5%), the Mann–Whitney U test (significance level at 5%), and the Kruskal–Wallis test (significance level at 5%) as appropriate [26,27,28]. The statistical tests were employed for the data presented in Figure 1 and Figure 2, Section 3.2.1, Section 3.2.2, Section 3.2.4 and Section 3.3.3. No survey response(s) were excluded from the analyses. 

## 3. Results

A total of 40 patients at the University Hospital in Frankfurt were successfully recruited to complete the patient survey. Similarly, 15 physicians from the University Hospital Frankfurt, University Hospital Erlangen, and Charité Berlin participated in the physician survey. Finally, three industry representatives were included in the survey of industry representatives.

### 3.1. Participant Characteristics

The majority of participating patients (25%) were aged over 65 years, with a further 22.5% aged between 36 and 45 years. Furthermore, 20% of participating patients were aged between 56 and 65 years, 17.5% between 46 and 55 years, and 15% between 26 and 35 years (Table 1). The majority of participating physicians (40%) were aged between 26 and 35 years (Table 1), with a further 26.7% aged between 46 and 55 years. Furthermore, 20% of participating physicians were aged between 36 and 45 years and 13.3% were aged between 56 and 65 years (Table 1). The survey did not inquire about age for representatives of the pharmaceutical industry. Assistant physicians constituted the majority of the participating physicians (53%), followed by specialists (20%) and senior physicians (20%). Chief senior physicians comprised 7% of the participants (Table 1).

### 3.2. Patient Survey

#### 3.2.1. Experience with Digitization

The majority of patients (60%) reported consistent use of digital technologies in their daily lives (e.g., smartphones, tablets, smart watches, etc.), with 27.5% of patients indicating frequent use (Table 2). Among the experiences with digitization in healthcare, appointments (62.5%) and prescriptions (22.5%) were the most frequently mentioned (Table 2).

**Figure 3:**
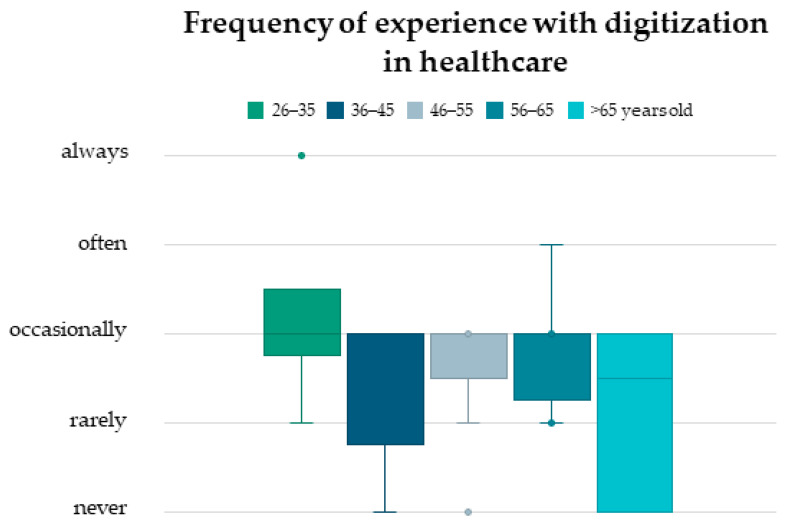
Boxplots showing the patients’ experience with digitization in healthcare (e.g., online appointments, electronic patient records, online consultations, health applications, etc.), broken down by age groups (green 26–35 years old, dark blue 36–45 years old, grey 46–55 years old, blue 56–65 years old, light blue > 65 years), which showed no significant differences between ages (*p* > 0.05).

The youngest age group (26–35) reported the highest frequency of experience with digitization in healthcare, with an average range from occasionally to often (Figure 3). In the older age groups, the average frequency ranged between occasionally and never, and no statistically significant differences were observed between age groups. 

Subsequently, patients were invited to define the concept of “patient-friendly digitization” in their own words. 

The issue of data security was raised by 12 out of the 40 patients surveyed, with specific references to the safeguarding of personal data and the protection of sensitive information. The benefits of timesaving and administrative support were identified by 10 out of the 40 patients, with additional comments pertaining to the aggregation of data from multiple healthcare providers and the expedited access to personal health information, including medical diagnoses, prescriptions, and referrals. The use of digital communication channels was mentioned by six patients, with discussions centering around the convenience of online appointments and referrals.

#### 3.2.2. Data Handling

**Figure 4:**
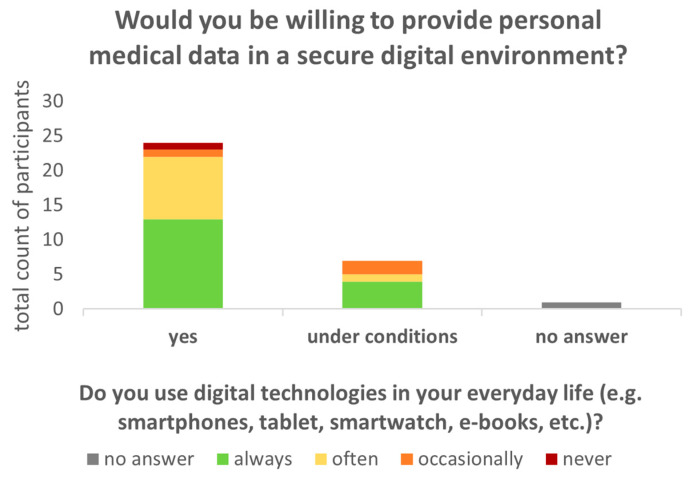
Readiness of patients to provide medical data in a secure environment (total count of participants on *y*-axis, willingness on *x*-axis) does not depend on the extent to which patients use digital technologies in their daily lives (color-coded stacks, *p* > 0.05). Provision of data is also independent of age (*p* > 0.05).

Patients who reported greater use of digital technologies in their everyday lives (always or often) were more inclined to provide personal medical data (Figure 4).

Upon inquiry as to the types of data patients would be amenable to sharing in a secure environment, respondents indicated a willingness to divulge personal data, organizational data, treatment information, and results, including imaging data. There was no discernible preference for specific data categories to be shared, even when patients expressed interest in clinical trials (*p* > 0.05).

#### 3.2.3. Digitized Communication with Physicians

Table 3 illustrates that 65% of patients expressed willingness to share data such as blood pressure or blood sugar levels digitally with their physician. Conversely, 25% of patients indicated disinterest in sharing this type of data digitally (Table 3). Similarly, 67.5% of patients indicated a willingness to engage in digital communication with their physician via a secure platform for the purpose of discussing medical data, whereas 25% expressed a lack of interest in doing so (Table 3). With regard to the digital management of medication, 50% of patients indicated a general interest, while 42.5% expressed no interest or satisfaction with the existing solutions. The majority of patients (70%) indicated a willingness to receive further information from their physician digitally regarding medication and therapy options (Table 3). With regard to data management, the majority of patients (45%) expressed a preference for pseudonymized data, 27.5% indicated a preference for anonymized data, and 20% of the patients were uncertain (Table 3).

Patients were asked to define what is meant by the term “data privacy/security” in a personal context. The following keywords were listed by the patients: SSL encryption, encryption similar to that used for bank accounts, data availability on premises (not cloud), email address authorization, two-fold authentication, and access restriction.

The majority of patients (82.5%) indicated a willingness to share data for the purpose of improving personal care, with an additional 72.5% expressing a similar willingness to contribute to improvements in general care (Table 4). With regard to the sharing of data for research purposes, the majority of patients (80.0%) indicated a willingness to do so with universities, followed by research institutes (67.5%), health insurance companies (42.5%), and industry (20%). Only 10% of patients reported no restrictions regarding the sharing of their data (Table 4).

The majority of patients (55.0%) indicated that they would expect enhanced medical care as a result of data sharing, while 47.5% stated that they would expect information regarding the utilization of the data. A mere 12.5% of respondents indicated that they would expect monetary compensation for their data (Table 4). With regard to the period of data access, 37.5% of patients would prefer to be consulted annually regarding the continuation of data usage, while 32.5% of patients would prefer to be consulted on a case-by-case basis for each individual request. Only 17.5% of patients would permit uninhibited access to their data (Table 4). With regard to the administration of access to their data, 47.5% of patients would prefer to exercise control over this matter, either by limiting access to specific data and/or a specified time period, or by allowing access to all data on a user-group basis. Conversely, 37.5% of patients would not wish to assume responsibility for managing access to their data (Table 4).

#### 3.2.4. AI in Healthcare

With regard to the question of whether patients felt adequately well informed about the potential applications of artificial intelligence (AI) in healthcare, no statistically significant differences were observed between the age groups (*p* > 0.05). The median response across all groups was “undecided” (five-point Likert scale: fully agree, slightly agree, undecided, slightly disagree, fully disagree).

Patients offered several potential benefits of AI usage in healthcare, including diagnostic support, appointment management, information on findings, cost-efficient treatment, and early detection of diseases (Table 5). Conversely, potential issues with AI in healthcare were also identified, including distrust (impersonal interaction), challenging error interpretation, lack of traceability of decisions, data loss, and misuse of data (Table 5).

**Figure 5:**
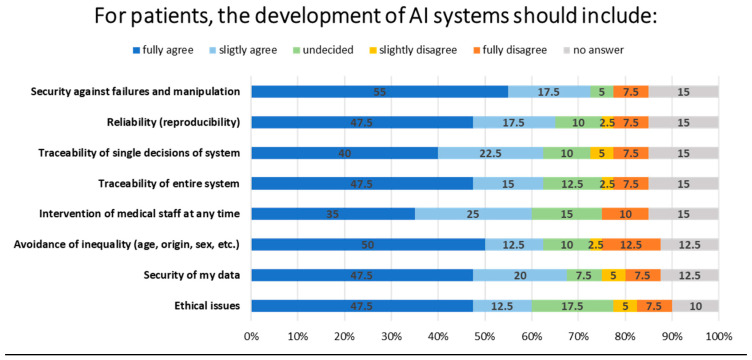
Sentiment overview barometer regarding the development of AI systems with medical data from patients. Percentages to which patients fully agree (dark blue), slightly agree (light blue), are undecided (green), slightly disagree (dark yellow), fully disagree (orange), or abstain (gray) from engaging with these statements.

The majority of patients (50% and 47.5%, respectively) identified the fair use of AI (e.g., no discrimination by gender, age, origin, etc.) and ethical issues as important considerations for the development of AI systems. The respondents indicated that the traceability of the entire system (47.5%), the security of data (47.5%), the possibility of intervention by physicians at any time (47.5%), reliability (47.5%), and security against failures and manipulation (55%) were the most important factors (Figure 5).

### 3.3. Physician Survey

#### 3.3.1. Experience with Digitization

In general, physicians expressed the view that digital applications could be beneficial in healthcare. Specifically, 60% indicated that they believed this to be the case, 26.7% suggested that this was probably right, and 13.3% expressed uncertainty. They also perceived potential across all areas, with 86.7% indicating that they believed digital applications could facilitate general communication between physicians, 86.7% suggesting that they could enhance communication within clinics on topics including imaging, medication schedules, laboratory values, and others, and 66.7% indicating that they could be useful in the research area, particularly in providing context on current and potential study participation.

The ideal digital patient overview, as identified by physicians, would comprise the patient master file (80%), laboratory values (73.3%), imaging (CT/MRI/US/etc.) (73.3%), and other data, such as medication history, allergies, and pre-treatments (33.3%).

In terms of the significance of graphical representations of parameters in clinical routine care, 40% of the physicians surveyed rated them as “fairly important”, while 6.7% deemed them “very important”. A total of 26.7% of the respondents were “undecided”, and only 20% stated that the graphical representation of specific parameters was “not important”.

#### 3.3.2. Need for Upcoming Digitization

A significant component of the questionnaire administered to physicians was for ascertaining their desired features and expectations of a prospective digital platform. 

The concept of usability was identified as a pivotal factor, with physicians citing key terms such as “easy to use”, “intuitive”, “easy handling”, “clarity”, and “overview” (of diagnoses, laboratory values, and medications). Another crucial aspect identified by the physicians was time efficiency. This encompassed several key issues, including effectiveness, rapid data transfer, accessibility of all data, and synchronization with other physicians’ visits. Additionally, data security was a prominent concern, encompassing topics such as patient safety, secure data transfer, and secure communication between employees. Furthermore, the physicians highlighted the importance of practice-oriented solutions, the replacement of existing platforms, and interoperability, emphasizing the need for a comprehensive, integrated platform. Furthermore, in order to ascertain the necessity for improvement and to ensure optimal time management, we requested estimated timeframes for patient visits. In the case of initial patients, medical practitioners estimated that approx. 20 min would be required for the documentation of patient data. In contrast, for subsequent follow-up visits, the estimated time was reduced to 11 min. 

In addition to the aforementioned expectations and wishes, we also inquired as to how a novel platform would need to differ from existing ones in order to create an additional benefit. 

The concept of integrative functionality was identified as a crucial element, with key terms such as “all programs in one app”, “interface connection”, “availability across all settings”, “communication/data exchange across hospitals”, “communication with health insurance companies and pension funds”, and “communication with private practices”. Another notable aspect was the emphasis on clear design, encompassing graphics and tables, highlighting crucial findings, providing a structured overview, and offering a quick overview. Additionally, the keywords “faster”, “easier”, “inclusion of assessments”, and “therapy forms” emerged as important considerations. In terms of processes that require automation in the context of patient data management, the physicians identified several potential candidates, including documentation of pre-existing conditions (either already documented within the system or otherwise), findings (history, imaging, laboratory), medication (history, changeover, current), and the creation of physicians’ letters and new findings.

Eight out of fifteen participants (chief senior physicians, senior physicians, specialists, and assistant physicians) considered that comparing individual patient data with cohorts was a valuable asset for diagnosis, while six out of fifteen participants (specialists and assistant physicians) remained undecided on this manner. One assistant physician expressed disagreement with this statement.

Additionally, the participants were queried regarding the information they would like to be able to utilize from a pool of patient data (data mining) to enhance the efficiency and quality of their daily routine care. The participants identified laboratory values and histories, medication/premedication, diagnoses, and findings (examination, imaging, scores) as information that they would like to use.

#### 3.3.3. AI in Healthcare

With regard to the question of whether physicians feel adequately informed about the potential applications of AI in healthcare, no significant difference was observed between the age groups (*p* > 0.05). The median response across all groups was “slightly disagree” (five-point Likert scale: fully agree, slightly agree, undecided, slightly disagree, fully disagree).

**Figure 6:**
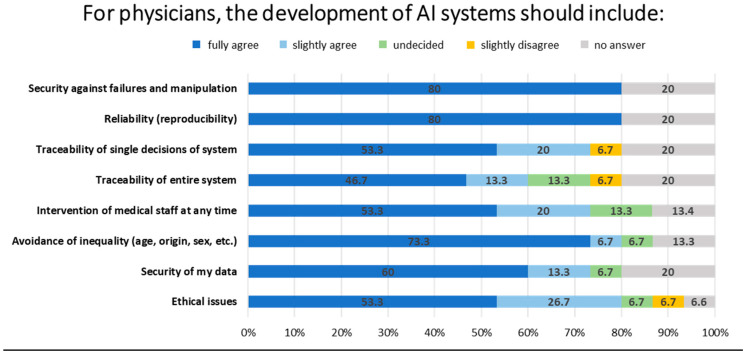
Overview sentiment barometer regarding the development of AI systems with medical data from physicians. Percentages of physicians whether they fully agree (dark blue), slightly agree (light blue), are undecided (green), slightly disagree (dark yellow), or abstain (gray) from engaging with these statements.

In the development of AI systems, 80% of medical participants indicated that reliability and security against failures and manipulation should be included. Additionally, 73.3% of participants identified the avoidance of inequality as a crucial element, while 60% emphasized the security of data as a vital component of AI systems (Figure 6).

The physicians provided examples of potential advantages of AI usage in healthcare, including the analysis of imaging, appointment management, drug interactions, prediction of disease progression, disease activity monitoring, and treatment decisions (Table 6). Conversely, they also outlined potential challenges associated with AI in healthcare, including a lack of transparency, dependency, further estrangement of patients, misdiagnosis, and misuse of data (Table 6).

### 3.4. Representatives of the Pharmaceutical Industry

Given the limited feedback from pharmaceutical representatives (*n* = 3), this paper only provides a brief overview of the most salient points without undertaking any statistical analyses.

The representatives of the pharmaceutical industry highlighted the significant potential of medical data for advancing research.

Furthermore, they highlighted that the current data protection regulations, particularly the General Data Protection Regulation (GDPR), are perceived as overly complex and stringent. Nevertheless, they acknowledge that data protection is a high priority, including data management and data sovereignty that lies with the patients.

Contacting participants for potential study participation and the time needed for recruiting and administrative management for clinical studies are crucial and often main (limiting) issues for the performance of clinical studies. Representatives of the pharmaceutical industry explained that the use of big data and AI would generally be desirable but is currently not feasible due to insufficient data and legal regulations.

In order to facilitate the development and implementation of novel platforms or applications, it was emphasized that it would be imperative to engage all stakeholders at the earliest possible stage of the process. Moreover, it was suggested that novel platforms could potentially facilitate the provision of information to patients participating in clinical studies, such as updates via push notifications. Additionally, the representatives of the pharmaceutical industry would find it beneficial if the aforementioned platforms were able to facilitate and oversee the withdrawal of consent and participation. In a similar vein, representatives of the pharmaceutical industry would perceive a distinct advantage if such a platform could furnish patients with prospective consent options for future studies (dynamic consent) and if a legally compliant integration and transmission of the consent within the eCRF were feasible. Additionally, they posited that another advantageous feature within a novel platform could be if the platform elucidated the benefits to patients from study participation beyond mere remuneration.

## 4. Discussion

Integrating applications into clinical routines will be critical for digital healthcare to reach its full potential [10]. More precisely, hospitals and care providers are already employing digital technologies, including artificial intelligence (AI), machine learning, smart sensors, and big data analytics, with the objective of enhancing the quality of care and operational efficiency [11]. The application of such technologies demonstrates considerable potential, ranging from relatively straightforward innovations in operational processes to the most challenging treatments of emergency patients [12]. Furthermore, policies and ethical guidelines for healthcare services emphasize the importance of obtaining informed consent, ensuring data security and management, and employing such technologies in a responsible manner [13]. To facilitate broad acceptance and targeted implementation of future systems, it is essential to involve stakeholders of (medical) data in a transparent process. 

This exploratory study aimed to gain initial insights into patient and physician perspectives on healthcare data management, data sharing, the concept of digitization in the healthcare sector, the implementation of novel methodologies such as AI, and the development of novel digital platforms. To this end, this exploratory study surveyed patients and physicians based on the aforementioned topics. Furthermore, the study aimed to identify potential benefits for and interests of patients and physicians in relation to the management of medical data in novel systems.

### 4.1. Patients

The utilization of digital technologies in the healthcare sector has proliferated across all age groups and is currently being employed in a multitude of domains (Figure 3).

The concept of patient-friendly digitization encompasses a range of features, including data security, timesaving and administrative support, and digital communication. These elements are perceived as beneficial by patients, along with additional aspects such as data protection, rapid access to data, and online administration. These attributes extend beyond the domain of medical care, reflecting a broader perspective on the potential of digital technologies in healthcare. Patients have identified the creation of an application that provides already familiar standards (such as data security) and functionalities (such as digital overview and management of diagnoses, prescriptions, referrals, and appointment bookings) as a priority.

The willingness to provide medical data in a digital environment exhibited a range of responses, from affirmative to reserved (“yes” or “under conditions”). However, this willingness did not appear to be contingent on the extent to which patients utilized digital technologies in their daily lives (Figure 4). Overall, it is notable that none of the patients expressed a complete rejection of the concept of sharing their medical data. Rather they indicated either agreement or partial agreement, contingent upon specific conditions such as maintaining anonymity or limiting the scope of data shared to certain information.

The digitization of communication with physicians represents a significant challenge for emerging medical platforms and applications (Table 3). In particular, the digital transmission of information on a frequent basis (e.g., blood pressure measurements or blood glucose levels) could present a significant time saving in everyday life. The necessity for in-person visits to the physician for the purpose of taking measurements that could be taken at home could be reduced, thus improving time management for patients. The same is true of consultations with the treating physician—for example, regarding therapy and medication options. Digital solutions have the potential to be employed in the context of queries, initial assessments, or even discussions of findings, thereby supplementing personal visits in a time-efficient manner. A mean of 67.5% of patients indicated interest in digital solutions in this regard, while only 22.5% expressed skepticism, citing the lack of technical possibilities or expense (Table 3). However, all transmitted values must be viewed and evaluated by physicians, which in turn can be time-consuming and would require appropriate software.

In the context of medical data, data protection is of particular importance. It was thus our initial intention to ascertain the manner in which patients conceptualize the notion of data protection or data privacy (free text option). Two key points emerged from the data: Firstly, a number of patients with a high level of digital literacy proposed specific solutions, including the use of SSL encryption, encryption methods similar to those employed by financial institutions, and two-factor authentication. Conversely, a considerable number of patients elected to leave the text field blank, thereby abstaining from offering any suggestions whatsoever. This could be attributed to a dearth of data literacy or a paucity of interest with respect to this subject matter [29]. Consequently, it is imperative to accord particular attention to the enhancement of data literacy, the clarification of privacy (anonymous vs. pseudonymous) in straightforward terms, and the promotion of data encryption.

The results of our survey on data sharing and management indicate that patients are more likely to provide data for the purpose of enhanced medical care (77.5%) than for the purpose of supporting research (47.5%). The discrepancy in results may be attributed to the fact that our survey was conducted with patients who are currently experiencing the adverse effects of their underlying disease(s). It is plausible that their preference for an immediate improvement in medical care over the future advancement of research may have influenced the outcome. In the context of (applied) research, patients expressed a clear preference for universities (80%) and known research institutes (67.5%) as trustees of their medical data, in contrast to health insurers (42.5%) and the pharmaceutical industry (20%)—findings that align with those of other studies [30,31]. This suggests the necessity for transparency regarding data utilization, encompassing transparent access controls, clear delineation of permitted topics, and timeframes. Additionally, there is an opportunity to foster trust through effective communication. Patients are more likely to share their medical data if they trust that they will be used for enhanced healthcare and to inform future developments, rather than for monetary gain (Table 4).

A further key element of the questionnaire was the patients’ views on the potential role of AI in healthcare. It is notable that no significant differences were observed with regard to age groups, with all patients reporting similar levels of awareness and understanding of AI. However, there was considerable variation in the level of knowledge demonstrated. The median response across all groups was “undecided” regarding their level of knowledge, which may indicate a dearth of accessible and intelligible information on this subject matter for the general public. The use of free text enabled patients to identify and emphasize the key advantages and disadvantages associated with the deployment of AI in healthcare. Notably, the participating patients demonstrated a comprehensive understanding of the advantages of AI in healthcare, including facilitating early diagnosis, acquiring supplementary information (such as medical reports), and providing cost-effective care and scheduling visits. The responses to the question regarding disadvantages also revealed a number of concerns, including the potential for false interpretation by AI, lack of traceability, loss or misuse of data, loss of control, and the transfer of personal data into a black box (Table 5). Once more, the results indicate that the dissemination of targeted information to patients is important, as is the communication of the methods employed by AI in the analysis of medical data. The utilization of machine learning, text mining, or neural networks typically represents a subject matter with which only a limited proportion of the population is familiar. Nevertheless, when prompted to specify the desired characteristics of AI utilization in medical data, patients exhibited a discernible preference. On average, 60% or more of respondents endorsed the adherence to and assurance of specific medical standards, data protection standards, and ethical standards throughout the development and deployment of AI in healthcare (Figure 5).

### 4.2. Physicians

The majority of the participating physicians indicated that digital applications would provide benefits, with 60% responding affirmatively and 26.7% expressing a probable affirmative response. The remaining votes were cast in favor of an “I don’t know” view regarding this topic (13.3%). No participant expressed the opinion that there would be no benefit. This suggests that, across the age groups within the group of participating physicians, there is an awareness of digitization and its role in healthcare. It is evident that digitization has been a significant factor in the field of healthcare for some time. Nevertheless, the fact that further potential is still being seen demonstrates that the evolving character of digitization fosters ongoing changes that require attention. However, we still face a lack of sufficient and effective platforms in daily routine care. Participating physicians discerned potential areas for improvement across a spectrum of domains, including general communication between physicians (86.7%), clinical communication pertaining to imaging, drug schedules, laboratory values, and so forth (86.7%), as well as clinical research with context on current or prospective study participation (66.7%).

Based on the physicians’ responses, it might be recommendable that future digitized platforms or applications prioritize usability, time efficacy, and data security. In addition, interoperability was explicitly mentioned. This illustrates a distinctive feature that has historically presented challenges to physicians and continues to do so presently. This feature is the availability of numerous digital platforms that are incompatible with one another [32].

In terms of expectations for future digital platforms, there is an overlap with aspects that also appear to be favored by patients, namely usability, time management, data security, and digital communication. This suggests the potential for combining the needs of different stakeholders in a single system, with the caveat that individual issues may require targeted solutions.

It is important to consider that medical staff have indicated that a first patient visit typically requires approximately 20 min (with a follow-up visit requiring approximately 11 min). This indicates the critical importance of implementing time-efficient data management strategies, as the majority of the allocated time should be dedicated to patient care. These figures are comparable to those previously reported. In the United States, 45% of rheumatologists stated that they typically spend 17–24 min with a patient, while 32% of rheumatologists reported spending 13–16 min with a patient [33,34]. 

The importance of a customized, user-friendly interface and extensive data accessibility is particularly evident when identifying the “important core data of patients” required by physicians. This encompasses the patient master file (80%), laboratory values (73.3%), and imaging (including CT, MRI; US, 73.3%). Furthermore, the majority of participating physicians identified the visualization of parameters as being either “very important” (6.7%) or “important” (40%). Interestingly, physicians identified similar topics when asked to describe the desired functionality of a future platform, as compared to existing ones. These included integrative structures (such as an all-in-one application, comprehensive access, and communication with health and pension insurance or private practices, assessments), as well as clarity (such as a concise overview, graphical representations and tabular data, and patient reports). In response to the question of which data they would like to be able to filter out from the pool of patient data (data mining) for a more efficient daily routine in treatment, the respondents indicated a high degree of redundancy in their responses, citing items mentioned previously, such as laboratory values and histories, medications/premedication, diagnoses, and findings (examination, imaging, scores). In this context, the relevance of the data was seen to extend beyond their use in analyses, with the possibility of automated data management also being highlighted. These data suggest the necessity for enhanced time management (given the vast quantity of data) and the improvement of medical care methodologies. Additionally, all participating physicians concur that the evaluation of patients in cohorts or the comparison of individual patients with such cohorts is a valuable addition to the diagnostic process.

In order to meet the aforementioned requirements (pattern recognition, summarization of large amounts of data, image analysis, etc.), novel approaches for data analysis could be employed, including the use of AI. Interestingly, the majority of participating physicians expressed uncertainty regarding their level of knowledge about AI or indicated a lack of familiarity with the subject matter. Nevertheless, it seems reasonable to posit that this represents a deficit in terms of the potential methods that could be employed (for example, machine learning, neural networks, and pattern recognition) and their implementation in systems (in terms of software and programming). With regard to the standards for the utilization and deployment of AI, the participants identified some ideas pertaining to regulations and safety. There was a markedly higher level of consensus among the participating physicians compared to the participating patients, with over 60% and 70%, respectively, agreeing on the importance of data security and traceability, and 80% on the reproducibility of results when developing AI systems (Figure 6). The physicians identified several potential advantages of AI in clinical practice, including the analysis of imaging data (e.g., histology, radiology), drug interaction, prediction of disease progression and disease activity monitoring, and treatment decision-making. On the other hand, they also highlighted several potential challenges associated with the use of AI, including lack of transparency, dependency on specific systems or software, further estrangement from patients, and misdiagnosis (Table 6).

### 4.3. Study Limitations

The results are exploratory in nature and not generally representative due to the small number of completed surveys (*n* = 40 for patients and *n* = 15 for physicians). Furthermore, the inclusion of patients only within a single indication area (rheumatology) and from a single hospital (University Hospital Frankfurt) introduces another limitation, as views of patients with different diseases or conditions may differ from those with rheumatological conditions. 

A limitation of the study is that the responses from physicians were obtained from only three university hospitals, all of which were within the field of rheumatology. With regard to the patients, it is possible that physicians from other specialties may hold divergent views and possess different experiences regarding digitalization within the healthcare sector. Furthermore, physicians who are not employed in university hospitals, for instance those working in small private practices, may also have varying perspectives and experiences, given that the working environment is likely to differ. Consequently, the findings of this exploratory study would benefit from being expanded to encompass other indications, additional hospitals, and locations outside of university hospitals, in order to achieve a more representative character.

## 5. Conclusions

The present exploratory study examines certain aspects of patients’ and physicians’ perceptions of digitalization in the healthcare sector, as well as their attitudes towards the handling of medical data.

The findings of this exploratory study indicate that data security, timesaving/administrative support, and digital communication are aspects that patients associate with patient-friendly digitization. This includes additional items such as data security, data protection, fast access to data, and online administration, which are areas of digitization beyond medical care. Patients emphasize the development of an app that provides already familiar standards and functionalities. Furthermore, the data from this exploratory study indicate that patients require transparency regarding data use, as well as the opportunity for clear communication and the establishment of trust. Based on the survey results, the patients’ trust in the use of their medical data for enhanced healthcare and the reflection of results is a greater motivator for data sharing than monetary benefits.

The results indicate the necessity for an enhanced approach to time management for clinicians, coupled with the implementation of advanced medical care methodologies. In light of the physicians’ responses, it is recommended that future digital platforms or applications prioritize usability, time efficiency, and data security. Furthermore, the term “interoperability” was explicitly referred to. To fulfill some of the aforementioned requirements, innovative approaches to data analysis could be employed, including the use of AI.

With regard to expectations for future digital platforms, there is a notable overlap between the expectations of patients and physicians, particularly in terms of usability, time management, data security, and digital communication. This suggests that the needs of different stakeholders can be integrated into a unified system, while still allowing for the development of targeted solutions to specific issues.

## Figures and Tables

**Table 1:** Overview of the characteristics of the survey participants. N = number.

Variable	Patients (*n* = 40) N (Relative Frequency, %)	Physicians (*n* = 15) N (Relative Frequency, %)	Representatives of the Pharmaceutical Industry (*n* = 3)
Age:			
18–25	0 (0.0)	0 (0.0)	The pharmaceutical survey did not include a question regarding age.
26–35	6 (15.0)	6 (40.0)	
36–45	9 (22.5)	3 (20.0)	
46–55	7 (17.5)	4 (26.7)	
56–65	8 (20.0)	2 (13.3)	
>65	10 (25.0)	0 (0.0)	
Current position:	-		-
Executive senior physician		1 (6.7)	
Senior physician		3 (20.0)	
Specialist physician		3 (20.0)	
Assistant physician		8 (53.3)	

**Table 2:** Patients’ experience with digitization. The results are presented as absolute values (N) and relative frequencies (%). In the case of experience with digitization in healthcare, respondents were permitted to provide more than one answer.

Usage of Digital Technologies in Daily Life N (Relative Frequency, %)		Specification of Experiences with Digitization in Healthcare N (Relative Frequency, %)	
Never	1 (2.5)	Appointments	25 (62.5)
Occasionally	3 (7.5)	Prescriptions	9 (22.5)
Often	11 (27.5)	Healthcare/insurance applications	5 (12.5)
Always	24 (60.0)	Online consultations	2 (5.0)
No answer	1 (2.5)	Sick notes	2 (5.0)
		Electronic patient records	1 (2.5)

**Table 3:** Overview of questions about digital communication with physicians and corresponding patient responses. The results are presented as absolute values (N) and relative frequencies (%).

Question	Answer Options	Frequency of Patient Response N (Relative Frequency, %)
Would you be interested in sharing data like blood pressure or blood sugar digitally with your physician?	Yes	26 (65.0)
	No (I do not have the ability, effort too large)	10 (25.0)
	No (without reason)	4 (10.0)
Would you, in addition to the personal visit, communicate with your attending physician via a secure platform and discuss medical data?	Yes	27 (67.5)
	No (I do not have the ability, effort too large)	10 (25.0)
	No (without reason)	3 (7.5)
Would it be helpful for you to manage your current medication digitally, e.g., with the help of a weekly overview, reminder function, and notes that show the medication and dosage for each day?	Yes	20 (50.0)
	No (satisfied with existing possibilities)	17 (42.5)
	Already use such an offer No answer	1 (2.5) 2 (5.0)
Would you like your physician to provide you with further information regarding therapy and medication options?	Yes (time is often too short on site, good opportunity to read up on the facts)	28 (70.0)
	No (at visits, I receive all the necessary information)	7 (17.5)
	No answer	5 (12.5)
What type of basic delivery of your data would you prefer?	Pseudonymous data ^1^	18 (45.0)
	Anonymous data ^1^	11 (27.5)
	Uncertain No answer	8 (20.0) 3 (7.5)

^1^ Definition provided in questionnaire.

**Table 4:** Selection of questions about data sharing and data access and corresponding patient responses. The results are presented in numerical form (N) and as relative frequencies (%). For the first three questions, respondents were permitted to provide more than one answer.

Question	Answer Options	Frequency of Patient Response N (Relative Frequency, %)
For what purpose would you be willing to share data? ^1^	Improvement of personal care	33 (82.5)
	Improvement of general care	29 (72.5)
	Support for practice-oriented (applied) research	19 (47.5)
	Support for basic research	19 (47.5)
	Monetary compensation	5 (12.5)
Who would you share your data with for research purposes? ^1^	Universities	32 (80.0)
	Research institutes ^2^	27 (67.5)
	Health insurance companies	17 (42.5)
	Industry (e.g., pharmaceutical industry)	8 (20.0)
	No restrictions	4 (10.0)
Would you expect anything in return for your data? ^1^	Improved medical care (e.g., additional benefits)	22 (55.0)
	Information regarding the use of the data	19 (47.5)
	Inclusion/offerings for clinical studies	11 (27.5)
	No	8 (20.0)
	Money	5 (12.5)
Would you like to define the period of data access?	Being actively asked once a year whether data may continue to be used	15 (37.5)
	For each request individually	13 (32.5)
	Unlimited access	7 (17.5)
	Defined for a certain number of years No answer	2 (5.0) 3 (7.5)
Would you be interested in managing access to your data by yourself?	No	15 (37.5)
	Yes, with filter functions (only certain data, time periods, user groups, etc.)	14 (35.0)
	Yes (access on a user group basis, but all data) No answer	5 (12.5) 6 (15.0)

^1^ Patients were able to choose multiple answers. ^2^ E.g., Max-Planck, Fraunhofer, Paul-Ehrlich, Helmholtz, Leibniz.

**Table 5:** Patients’ views on AI solutions and problems in healthcare.

Advantages of AI Solutions	Issues Due to Use of AI Solutions
Diagnosis support Appointment management Information (e.g., on findings) Cost-efficient treatment Research Early detection of diseases	Loss of personal contact between patient and physician Misinterpretations Incomprehensibility of decisions Social exclusion Loss of data Misuse of data

**Table 6:** Physicians’ perspectives regarding AI solutions and problems in healthcare.

Advantages of AI Solutions	Issues Due to Use of AI Solutions
Automated image analysis Diagnosis support Prediction of disease progression Disease activity monitoring Treatment decision Early detection and mortality	Lack of transparency Discrimination Dependency Further estrangement of patients Misdiagnosis Important aspects are overlooked Extra effort

## Data Availability

The original contributions presented in the study are included in the article/Supplementary Materials (Tables S1 and S2); further inquiries can be directed to the corresponding author.

## Supplementary Materials

The following supporting information can be downloaded at https://www.mdpi.com/article/10.3390/healthcare12202053/s1, Table S1: Patient Survey and Table S2: Physician Survey.
