# Supplementary material for: Sharing for Caring? A Patients’ and Clinicians’ View on Handling Personal Medical Data in the Context of Digitization: An Exploratory Study
Source: Healthcare (Basel). 2024 Oct 16;12(20):2053. doi: 10.3390/healthcare12202053 (PMC11507340; doi:10.3390/healthcare12202053)
Supplement: Supplementary file 1 [file healthcare-12-02053-s001.zip › healthcare-3201458-supplementary.pdf]

Supplementary Material Table S1

Patients (n=40)

Question	Answer options	relative frequency
How old are you?	18-25	0
	26-35	15
	36-45	22,5
	46-55	17,5
	56-65	20
	>65	25
Do you use digital technologies in your everyday life (e.g. smartphone, tablet, smartwatch, e-books, etc.)?	never	2,5
	rarely	0
	occasionally	7,5
	often	27,5
	always	60
	No Answer	2,5
If you have technical problems with, for example, your PC or mobile phone, can you solve them yourself?	never	5
	rarely	15
	occasionally	35
	often	32,5
	always	10
	No Answer	2,5
Have you already had experience with digital technologies in healthcare (online appointment scheduling, online consultations, electronic patient files, health apps, e-prescriptions, etc.)?	never	17,5
	rarely	17,5
	occasionally	55
	often	5
	always	2,5
	No Answer	2,5
If yes, which ones (e.g. appointment, prescription, sick note, etc)	Appointments	62,5
	Prescription	22,5
	Health care/insurance app	12,5
	Online consultation	5
	Sick note	5
	Electronic patient record	2,5
What does patient-friendly digitalization mean to you?	Data security	30
	Time saving, administrative support	25
	Digital communication	15
	Other verbatim answers	Application orientation
		Understanding
		User-friendly
		Easy access
		Intuitive

		more autonomy
In principle, would you be willing to provide your medical data in a secure digital environment?	Yes	80
	No	0
	Only under certain conditions	12,5
	no answer	7,5
What data would you be willing to share? (multiple choice)	Personal data (age, gender, contact details)	67,5
	Organizational data (treating doctors, last hospital stays)	80
	Treatment data (diagnoses, medication plan, operations)	72,5
	Detailed treatment data (examination results, e.g. from X-rays or blood tests)	65
	Other	0
For what purpose would you be willing to share data? (multiple choice)	Improving your personal care	82,5
	Improving general care	72,5
	Support for practice-oriented (applied) research	47,5
	Support for basic research	47,5
	Other	2,5
Who would you share your data with for research purposes? (multiple choice)	Research institutes (e.g. Max-Planck, Fraunhofer, Paul-Ehrlich, Helmholtz, Leibniz)	67,5
	Universities	80
	Industry (e.g. pharmaceutical industry)	20
	Health insurance companies	42,5
	Other (professional associations, patient organizations, etc.)	12,5
	No restrictions	10
If you provide your information, what information would you like to receive? (multiple choice)	No further information	7,5
	A detailed overview of what exactly is being done with my data	57,5
	Information about which research projects	57,5

	the data will be used for	
	Results of the research projects	60
	Other	0
Would you expect anything in return for your data? (multiple choice)	No	20
	Information about what will be done with the data	47,5
	Inclusion/offers for clinical trials	27,5
	Improved medical care (expansion of services/additional services)	55
	Money	12,5
	Other	0
How would you like to determine the period of your data provision?	Individually for each request (e.g. project).	32,5
	I would like to be actively asked once a year whether my data can still be used	37,5
	Fixed for a certain number of years	5
	Unlimited	17,5
	no answer	7,5
Would you be interested in managing access to your data yourself?	Yes, according to the all-or-nothing principle (release all data to a specific user group)	12,5
	No	37,5
	Yes, with detailed filter functions that make it possible to only release certain data (e.g. only certain information, time restrictions for use, restrictions on user groups, etc.)	35
	no answer	15
How much effort should such data management involve?	I don't care about the effort as long as I get some benefit from it	22,5
	A few hours a month	7,5
	A few hours per year	32,5
	I don't want to spend additional time managing my medical information	25

	no answer	12,5
What priority is protecting your own privacy when sharing data?	A very high priority	50
	A high priority	20
	A medium priority	15
	A low priority	0
	A very low priority	2,5
	I'm not sure	5
	no answer	7,5
Which type of basic data access to your data would you prefer: anonymous or pseudonymous?	Anonymous data access	27,5
	Pseudonymous data access	45
	Undecided	20
	no answer	7,5
If you compare the usefulness of the data with data protection, how would you weigh up:	I expect maximum data protection, even if the usefulness of the data for research can then become almost worthless	20
	I consider usefulness and data protection to be of equal importance	30
	I consider the usefulness of the data more important than data protection (e.g. if the data leads to advances in the treatment of serious diseases)	27,5
	I'm not sure	10
	no answer	12,5
Please indicate briefly how you ideally envisage encryption and/or securing your data (e.g. type of encryption, backup systems, access restrictions). Please feel free to give examples from everyday life (e.g. how to back up my smartphone pictures, like in the XY program)	Verbatim answers	SSL and secure encryption
		Data availability within the university, on premise, not cloud
		adequate password assignment
		Encryption similar to bank accounts
		Backup in my computer
		Password synchronization
		only email addresses authorized by me
		Access restrictions
		Backup
		encoding
		Two-factor authentication
		Face recognition program

Would you like further information from your doctor regarding therapy and medication options?	Yes, time is often short in practice, so I could find out more myself	70
	No, when I visit the doctor I receive all the information that is important to me	17,5
	no answer	12,5
Would you like the opportunity to compare yourself with other patient groups in a medical context?	Yes	55
	No	35
	no answer	10
Would you like the opportunity to make contact with self-help groups and/or patient organizations in order to network outside of medical care?	Yes	40
	No	50
	no answer	10
Would you be interested in digitally sharing data such as blood pressure or blood sugar with your doctor?	Yes	65
	No	25
	no answer	10
If not:	I don't have the ability to do this (no device that can do this)	10
	The effort seems too great to me	7,5
	I don't trust technology	0
	Other	2,5
In addition to the personal visit, would you communicate with your treating doctor via a secure platform (e.g. app on PC, tablet, smartphone) and subsequently also discuss medical data (e.g. visualization of laboratory parameters, comparisons to groups of patients with the same disease , treatment options)?	Yes	67,5
	No	25
	no answer	7,5
If not:	I don't have the ability to do this (no device that can do this)	10
	The effort seems too great to me	5
	I don't trust technology	0
	Other	2,5
Would it be helpful for you to manage your current medication digitally, for example with the help of a weekly overview, reminder function and notes that show medication and dosage for each day?	Yes, that would make everyday life easier	50
	No, I am happy with the options I have for this	42,5
	I already use such an offer	2,5
	no answer	5

How do you rate the following statement: It would be a useful function for measurements taken at home (e.g. long-term ECG and blood pressure measurements) if these data were automatically transmitted to the treating doctor.	I fully agree	55
	I slightly agree	22,5
	Undecided	7,5
	I slightly disagree	2,5
	I fully disagree	2,5
	no answer	10
Would you be interested in an overview of currently ongoing studies on your clinical picture?	Yes	62,5
	No	27,5
	no answer	10
If yes, with what goal (multiple choice) 1 2	To be informed about the latest research results	50
	To take part in a study or to facilitate participation	30
	In order to follow the current course of the study when participating in a study (current status, next dates, etc.)	22,5
I feel sufficiently well informed about the possible uses of artificial intelligence (AI) in the healthcare sector.	I fully agree	7,5
	I slightly agree	12,5
	Undecided	37,5
	I slightly disagree	12,5
	I fully disagree	17,5
	no answer	12,5
In principle, I would be willing to make my data available for the development of AI systems that lead to better diagnosis or treatment of diseases.	Yes	65
	No	25
	no answer	10
In principle, I would be willing to make my data available for the development of AI systems that lead to more cost-effective treatments.	Yes	67,5
	No	25
	no answer	7,5
If I make my data available for the development of AI systems, it is important to me that ethical concerns are taken into account.	I fully agree	47,5
	I slightly agree	12,5
	Undecided	17,5
	I slightly disagree	5
	I fully disagree	7,5
	no answer	10
If I make my data available for the development of AI systems, it is important to me that my information is effectively protected.	I fully agree	47,5
	I slightly agree	20
	Undecided	7,5
	I slightly disagree	5
	I fully disagree	7,5
	no answer	12,5

If I make my data available for the development of AI systems, it is important to me that the system does not lead to unfair discrimination in terms of gender, age, origin or economic factors.	I fully agree	50
	I slightly agree	12,5
	Undecided	10
	I slightly disagree	2,5
	I fully disagree	12,5
	no answer	12,5
If I make my data available for the development of AI systems, it is important to me that medical staff can fundamentally intervene in the system and be given appropriate scope for action.	I fully agree	35
	I slightly agree	25
	Undecided	15
	I slightly disagree	0
	I fully disagree	10
	no answer	15
If I make my data available for the development of AI systems, it is important to me that the behavior of the system as a whole is understandable for me.	I fully agree	47,5
	I slightly agree	15
	Undecided	12,5
	I slightly disagree	2,5
	I fully disagree	7,5
	no answer	15
If I make my data available for the development of AI systems, it is important to me that I can understand the system's individual decisions.	I fully agree	40
	I slightly agree	22,5
	Undecided	10
	I slightly disagree	5
	I fully disagree	7,5
	no answer	15
If I make my data available for the development of AI systems, it is important to me that the results are reliable and repeatable.	I fully agree	47,5
	I slightly agree	17,5
	Undecided	10
	I slightly disagree	2,5
	I fully disagree	7,5
	no answer	15
If I make my data available for the development of AI systems, it is important to me that the system is secured against failures and attempts at manipulation.	I fully agree	55
	I slightly agree	17,5
	Undecided	5
	I slightly disagree	0
	I fully disagree	7,5
	no answer	15
In which specific areas do you hope AI solutions in the healthcare sector will be most beneficial?	Verbatim answers	Supporting diagnoses
		Make appointments
		Send information/findings
		cost-effective treatments
		Research
		Early detection of diseases
	none	7,5

What dangers do you personally see when using AI solutions in the healthcare sector?	Loss of personal contact between doctor and patient	7,5
	Other verbatim answers	uncertainty
		mistrust
		minor misinterpretations
		incomprehensibility of decisions
		espionage
		social exclusion of ill persons
		data loss
		misuse of data

Supplementary Material Table S2

Physicians (n=15)		
Question	Answer options	relative frequency
How old are you?	18-25	0,0
	26-35	40,0
	36-45	20,0
	46-55	26,7
	56-65	13,3
	>65	0,0
What is your current position?	medical director	0,0
	chief physician	0,0
	executive senior physician	7
	senior physician	20,0
	specialist physician	20,0
	assistant physician	53
	practical year student	0,0
If you have technical problems with, for example, your PC or mobile phone, you can:	Almost always solve it yourself	46,7
	Sometimes solve it yourself	46,7
	Almost never solve it yourself	6,7
	no answer	0,0
How do you rate safe and patient-friendly digitalization in the healthcare sector?	unimportant	0,0
	not that important	6,7
	I can't decide	6,7
	rather important	26,7
	very important	60,0
	no answer	0,0
	Yes definitely	60,0
	Probably yes	26,7

Can you generally imagine that you can benefit from a digital application/platform?	I don't know it	13,3
	Probably not	0,0
	No, not at all	0,0
	no answer	0,0
If yes, in which areas (multiple choice):	General: e.g. contact of treating doctors, last hospital stay	86,7
	Clinic: e.g. current imaging/medication plan/lab values	86,7
	Research: e.g. current/potential study participation	66,7
What do you wish from such an application/platform (multiple entries possible)?	Usability	66,7
	Time efficiency	33,3
	Data security	20,0
	Other verbatim answers	all data available
		practical
		replace other platforms
		synchronization with other doctor's visits
		interoperability
		fast data protection-compliant transmission
		All in one
		patient information tools
		exchange of information between all employees
		data sovereignty for patients
		overview of: diagnoses, laboratory values, medications, imaging
Do you already use other digital platforms/systems to supplement your own documentation, for example to simplify communication or ensure transparency (e.g. ePA)?	No	53,3
	Yes	40,0
	no answer	6,7
In which documentation systems do you primarily enter patient data?	Orbis	53,3
	SAP	13,3
	Xvianova	13,3
	Principa	13,3
	Metavision	6,7
What systems do your MFAs primarily use?	Orbis	40,0
	SAP	20,0
	Xvianova	6,7
	Principa	6,7
How much time do you have on average to document patient data?	First visit: mean: 20 minutes<br>standard deviation: 9 minutes	
	Follow-up visit: mean: 11 minutes	

	standard deviation: 8 minutes	
Which parameters would be essential for you in a central overview of the patient? (multiple choice)	master file	80,0
	laboratory	73,3
	Imaging (CT/MRI/US/others)	73,3
	Other	33,3
How important is a graphical representation of certain parameters to you?	unimportant	0,0
	hardly important	20,0
	I can't decide	26,7
	pretty important	40,0
	very important	6,7
	no answer	6,7
How does a digital platform have to differ from existing systems in order to offer additional value (multiple entries possible)?	Integrative in function	53,3
	Clear design	33,3
	User-friendly	26,7
	Other verbatim answers	faster
		variable data transmission
		integration of assessments
		DGRh therapy forms
Digital access to medical records from colleagues makes sense in everyday clinical practice.	I fully agree	100,0
	I slightly agree	0,0
	Undecided	0,0
	I slightly disagree	0,0
	I fully disagree	0,0
	no answer	0,0
The digital retrieval of all concomitant medications with a time period would be helpful in the anamnesis and treatment of patients.	I fully agree	93,3
	I slightly agree	6,7
	Undecided	0,0
	I slightly disagree	0,0
	I fully disagree	0,0
	no answer	0,0
If yes, in what time scale (multiple choice):	Last month	6,7
	Last quarter	0,0
	Last year	26,7
	Overall history	26,7
A digital platform for an overview of patient consents in the context of clinical studies would be useful (across centers).	I fully agree	46,7
	I slightly agree	26,7
	Undecided	26,7
	I slightly disagree	0,0
	I fully disagree	0,0
	no answer	0,0
An automated, daily transmission of diagnostic and treatment data in the outpatient area would be an effective	I fully agree	80,0
	I slightly agree	6,7
	Undecided	13,3
	I slightly disagree	0,0

way to accelerate/improve analyzes (of samples) and patient care.	I fully disagree	0,0
	no answer	0,0
Digital transmission of patients' self-recorded data, such as blood pressure or blood sugar, would usefully complement personal visits.	I fully agree	46,7
	I slightly agree	33,3
	Undecided	13,3
	I slightly disagree	6,7
	I fully disagree	0,0
	no answer	0,0
The ability to compare individual patients with a cohort would be a valuable addition to a diagnosis.	I fully agree	33,3
	I slightly agree	20,0
	Undecided	40,0
	I slightly disagree	6,7
	I fully disagree	0,0
	no answer	0,0
Are you currently exchanging patient data with other universities, clinics, institutes or pharmaceutical companies for research purposes?	Yes	26,7
	No	66,7
	no answer	6,7
I think exchanging current patient data digitally with other institutes, universities, clinics and pharmaceutical companies is complicated.	I fully agree	46,7
	I slightly agree	20,0
	Undecided	13,3
	I slightly disagree	0,0
	I fully disagree	6,7
	no answer	13,3
What would have to be changed/improved to make digital data exchange easier?	Nothing needs to be changed/improved	0,0
	things related to data protection	46,7
	Other	33,3
Would you use a GDPR-compliant option for easy communication with your colleagues (e.g. messenger, digital notes, appointment/service comparisons)?	Yes, that would make everyday work easier	93,3
	No, existing communication options are sufficient	0,0
	no answer	6,7
What do you think the start (rollout) for a patient data application/platform should look like?	Detailed workshop (possibly lasting several days).	13,3
	Half-day introduction and handout	46,7
	Online training video/website	20,0
	Handout and support via email and/or video chat	13,3
	no answer	6,7
	I fully agree	33,3

The possibility of independently creating documentation areas in an application/platform or personally adapting existing areas would optimize everyday use of it?	I slightly agree	46,7
	Undecided	6,7
	I slightly disagree	6,7
	I fully disagree	0,0
	no answer	6,7
In your opinion, the following existing systems should be linked to each other via an interface and in a future digital platform (e.g. DICOM / PACS / laboratory systems / intranet / PDMS / etc.):	Verbatim answers	PACS
		laboratory
		DICOM
		Lauris
		PACS
		All mentioned
		Interface between SAP+PACS
		DICOM, PACS, Lauris
What information would you like to be able to filter out/crystallize from the pool of patient data (data mining) in order to make your daily treatment routine more efficient or better?	medication/pre-medication	26,7
	Laboratory values/progressions	26,7
	diagnoses	13,3
	images	26,7
	Other verbatim answers	vital signs
		therapy-dependent activity scores
		therapy
		examination findings
		data points from anamnesis
In your opinion, which processes should be automated when managing patient data (e.g. because information already exists in another system / or has to be entered by hand)?	Medication	26,7
	Findings	60,0
	Other verbatim answers	Telephone/email availability
		Rehabilitation stays
		Physician's letter
Would an overview of the studies/projects in which the patient has taken part/is taking part be helpful in order to plan study examinations to be carried out and to be able to coordinate further studies/projects accordingly (adherence to maximum volumes when taking blood samples, etc.)?	Yes	60,0
	No	13,3
	no answer	26,7
Do you think that patients should have access to all data generated during treatment?	Yes	26,7
	No	60,0
	no answer	13,3
I feel sufficiently well informed about the possible uses of artificial intelligence (AI) in the healthcare sector.	I fully agree	0,0
	I slightly agree	6,7
	Undecided	20,0
	I slightly disagree	53,3
	I fully disagree	13,3

	no answer	6,7
When developing and deploying AI systems in healthcare, it is important to address ethical concerns.	I fully agree	53,3
	I slightly agree	26,7
	Undecided	6,7
	I slightly disagree	6,7
	I fully disagree	0,0
	no answer	6,7
When developing and using AI systems in the healthcare sector, it is important that the information processed is effectively protected.	I fully agree	60,0
	I slightly agree	13,3
	Undecided	6,7
	I slightly disagree	0,0
	I fully disagree	0,0
	no answer	20,0
When developing and using AI systems in the healthcare sector, it is important to ensure that there are no unfair discriminations in terms of gender, age, origin or economic factors.	I fully agree	73,3
	I slightly agree	6,7
	Undecided	6,7
	I slightly disagree	0,0
	I fully disagree	0,0
	no answer	13,3
When developing and using AI systems in the healthcare sector, it is important to ensure that medical staff can fundamentally intervene in the system and are given appropriate scope for action.	I fully agree	53,3
	I slightly agree	20,0
	Undecided	13,3
	I slightly disagree	0,0
	I fully disagree	0,0
	no answer	13,3
When developing and using AI systems in the healthcare sector, it is important that the behavior of the system as a whole is understandable for medical staff.	I fully agree	46,7
	I slightly agree	13,3
	Undecided	13,3
	I slightly disagree	6,7
	I fully disagree	0,0
	no answer	20,0
When developing and using AI systems in the healthcare sector, it is important that the system's individual decisions are understandable for medical staff.	I fully agree	53,3
	I slightly agree	20,0
	Undecided	0,0
	I slightly disagree	6,7
	I fully disagree	0,0
	no answer	20,0
When developing and using AI systems in the healthcare sector, it is important that the results are reliable and reproducible.	I fully agree	80,0
	I slightly agree	0,0
	Undecided	0,0
	I slightly disagree	0,0
	I fully disagree	0,0
	no answer	20,0
When developing and using AI systems in the healthcare sector, it is important	I fully agree	80,0
	I slightly agree	0,0

that the system is secured against accidents and attempted manipulation.	Undecided	0,0
	I slightly disagree	0,0
	I fully disagree	0,0
	no answer	20,0
In which specific areas do you hope AI solutions in the healthcare sector will be most beneficial?	Verbatim answers	Drug interaction
		Prediction of disease progression
		early detection, mortality
		Disease activity monitoring
		Therapy decision
		diagnosis
		Automated image analysis
What dangers do you personally see when using AI solutions in the healthcare sector?	Verbatim answers	Lack of transparency
		Discrimination
		Dependency
		extra effort
		growing estrangement
		incorrect diagnoses
		Important things are overlooked
